# Evaluation of right ventricular function in patients with pulmonary arterial hypertension by different right ventricular-pulmonary artery coupling methods

**DOI:** 10.1097/MD.0000000000030873

**Published:** 2022-09-30

**Authors:** Yaling Dong, Yu Li, Laichun Song

**Affiliations:** a Department of Cardiology, Wuhan Asia Heart Hospital, Wuhan, PR China.

**Keywords:** cardiac magnetic resonance, pulmonary artery hypertension, right heart function, single beat estimation, volume method

## Abstract

To compare the accuracy of end-systolic elasticity (*E*_es_)/arterial elasticity (*E*_a_) ratio measured by single beat estimation, pressure–volume loop and cardiac magnetic resonance (CMR) combined volume method in patients with pulmonary artery hypertension, and to find a feasible and reliable method to quantitatively evaluate the function of right ventricle in patients with pulmonary artery hypertension. Forty-nine pulmonary artery hypertension patients enrolled between May 2017 and May 2018 in our hospital were retrospectively analyzed. Firstly, measure *E*_es_/*E*_a_ ratio by single beat estimation, pressure–volume loop and CMR combined volume method, then, compare *E*_es_/*E*_a_ ratio with New York Heart Association (NYHA) classification and NT-proBNP value respectively to evaluate the accuracy of the 3 methods. *E*_es_/*E*_a_ ratio measured by single beat estimation is 2.07 ± 1.01, correlation analysis is not statistically significant when compare with NYHA classification and NT-proBNP value (*P* > .05). *E*_es_/*E*_a_ ratio measured by pressure–volume loop is 2.64 ± 1.48, correlation analysis is not statistically significant when compare with NYHA classification and NT-proBNP value (*P* > .05). *E*_es_/*E*_a_ ratio measured by CMR combined volume method is 0.72 ± 0.43, correlation analysis is statistically significant when compare with NYHA classification and NT-proBNP with negative correlation (*P* < .05). *E*_es_/*E*_a_ ratio decrease according to the increase of NT-proBNP value and the NYHA classification. There is linear regression equation between *E*_es_/*E*_a_ ratio measured by CMR combined volume method and log (NT-proBNP) value: *Y* = –0.257*X* + 1.45, and the linear regression equation is statistically significant (*P* = .001). *E*_es_/*E*_a_ ratio measured by CMR combined volume method is a feasible and reliable method to quantitatively evaluate the function of right ventricule in patients with pulmonary artery hypertension, which might be further verified in a larger patient population.

## 1. Introduction

The shape of the right ventricle in a normal subject is complex. Furthermore, its volume and surface area are difficult to measure owing to its complex geometry. The determination of right ventricle function and applicability of clinical parameters remains highly controversial. However, for patients with pulmonary arterial hypertension, accurate and reliable assessment of right ventricle function has a crucial predictive effect on clinical prognosis, and also provides important information for the development of reasonable and effective treatment programs for the patients.

The classification system of New York Heart Association (NYHA) is used for rapid clinical assessment of cardiac function status, and it is considered to be a good way for dividing the cardiac function status and predicting the prognosis of the patients by dichotomy.^[1,2]^ NT-proBNP is currently the optimal biochemical indicator for the diagnosis and management of heart failure.^[3,4]^ Currently, the classification system of NYHA and NT-proBNP are widely used in clinical practice to evaluate the function of the right heart in patients with pulmonary arterial hypertension. However, several studies^[5]^ have demonstrated that the gold standard method for assessing the right ventricle function in patients with pulmonary arterial hypertension must evaluate the contractility and after load. The right ventricle contractility can be quantified by maximal elasticity or the relationship between right ventricle pressure and volume, which are usually estimated using end-systolic elasticity (*E*_es_), or end-systolic pressure (ESP)/end-systolic volume (ESV). The most acceptable measurement of right ventricle afterload is arterial elasticity (*E*_*a*_), or ESP/SV. Multiple studies^[6,7]^ have shown that the right ventricle-pulmonary artery coupling (i.e., *E*_es_/*E*_a_) is an independent predictor of right ventricle function. The ideal *E*_es_/*E*_a_ ratio corresponding to the right ventricle-pulmonary artery coupling ranged approximately from 1.5 to 2, considering that the blood flow output is supplied with minimal energy consumption. Moreover, *E*_es_/*E*_a_ decreases as the pulmonary arterial pressure increases. Currently, there are 3 different methods for measuring *E*_es_/*E*_a_ clinically, which include single beat estimation, volume method and pressure–volume loop. The ratios of *E*_es_/*E*_a_ as measured by different methods vary. In our study, the right ventricle function of patients with pulmonary arterial hypertension was evaluated using single beat estimation, volume method and pressure–volume loop to find a practical and effective method for evaluating the function of right ventricle.

## 2. Materials and Methods

### 2.1. Study subjects

Forty-nine patients with pulmonary arterial hypertension who underwent treatment at Wuhan Asian Heart Hospital from May 2015 to May 2017 were retrospectively enrolled. Of the 49 patients, 14 were males and 35 were females, with an average age of 31 ± 12 years. All patients underwent right cardiac catheterization and were diagnosed with pulmonary arterial hypertension. All patients underwent 6-minute walking distance test and NT-proBNP test within one week, as well as cardiac magnetic resonance (CMR) within 2 weeks after cardiac catheterization. Pulmonary arterial hypertension was diagnosed according to the guidelines of American College of Cardiology in 2003. The mean pulmonary arterial pressure (mPAP) was ≥25 mmHg by previous or current cardiac catheterization. Patients with pulmonary and/or systemic vascular diseases were excluded. This study was reviewed and approved by the Ethics Committee of Wuhan Asian Heart Hospital. The patients signed the informed consent form.

### 2.2. Measurement method of the right ventricle-pulmonary artery coupling (*E*_es_/*E*_a_)

All enrolled patients underwent right cardiac catheterization under local anesthesia, and the right atrial pressure and pulmonary arterial pressure were recorded. The end-diastolic pressure (EDP) was recorded at the maximum diastolic pressure point before volumetric contraction, and ESP was recorded at the end of the systolic process.

#### 2.2.1. Single beat estimation.

**Figure 1.**
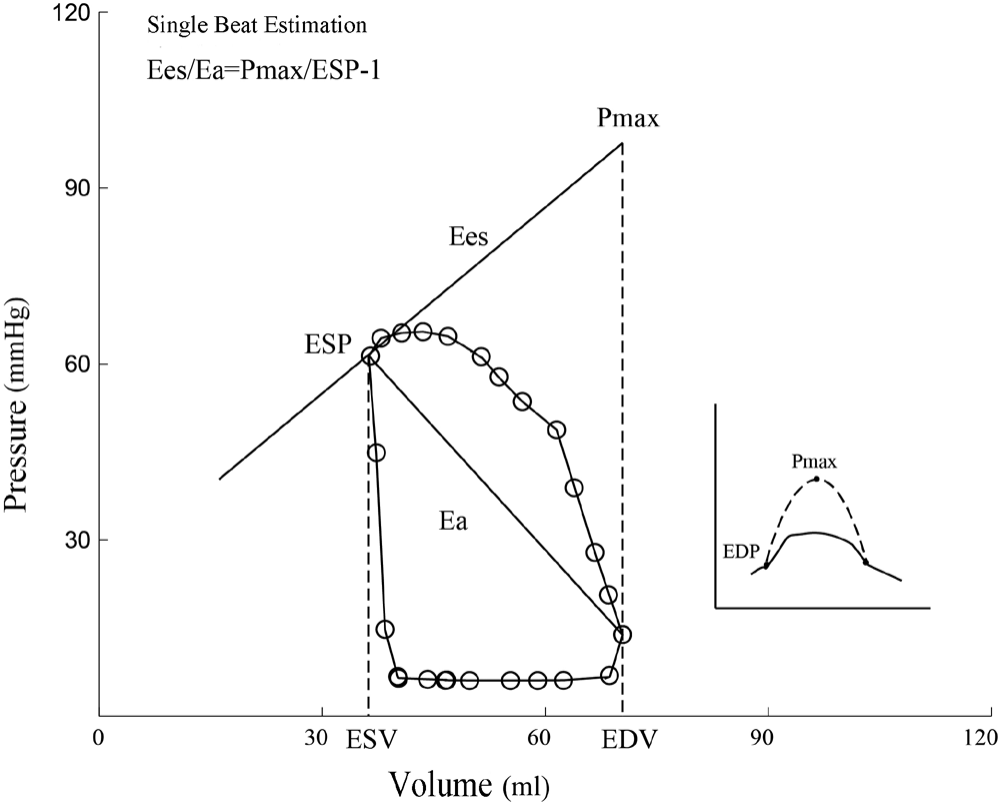
Measure *E*_es_/*E*_a_ ratio by single beat estimation. Ea = arterial elasticity, Ees = end-systolic elasticity.

Single beat estimate was first proposed by Takeuchi.^[8]^ In this method, evaluation was performed by individual pressure–volume loop obtained via the catheter, and the formula used was *E*_es_/*E*_a_ (S) = *P*_max_/*P*_es_ – 1. Of which, *E*_es_ was the ventricle elasticity at the end of ejection, which was defined as the slope of maximum isovolumic pressure (*P*_max_) at the end of systole, i.e. (*P*_max_ – ESP)/SV (Fig. 1). *P*_max_ was the maximum isovolumic pressure that was obtained by fitting a sinusoidal curve based on the right ventricle pressure waveform. *P*_max_ was estimated as the peak of the sine wave. *E*_a_ was the effective *E*_a_, and was defined as the slope of end-systolic point to the end-diastolic point on the pressure–volume loop. Supposing that the right ventricle ESP was 0, and then *E*_a_ can be estimated as ESP/SV.

#### 2.2.2. Volume method combined with CMR technique.^[9]^

**Figure 2.**
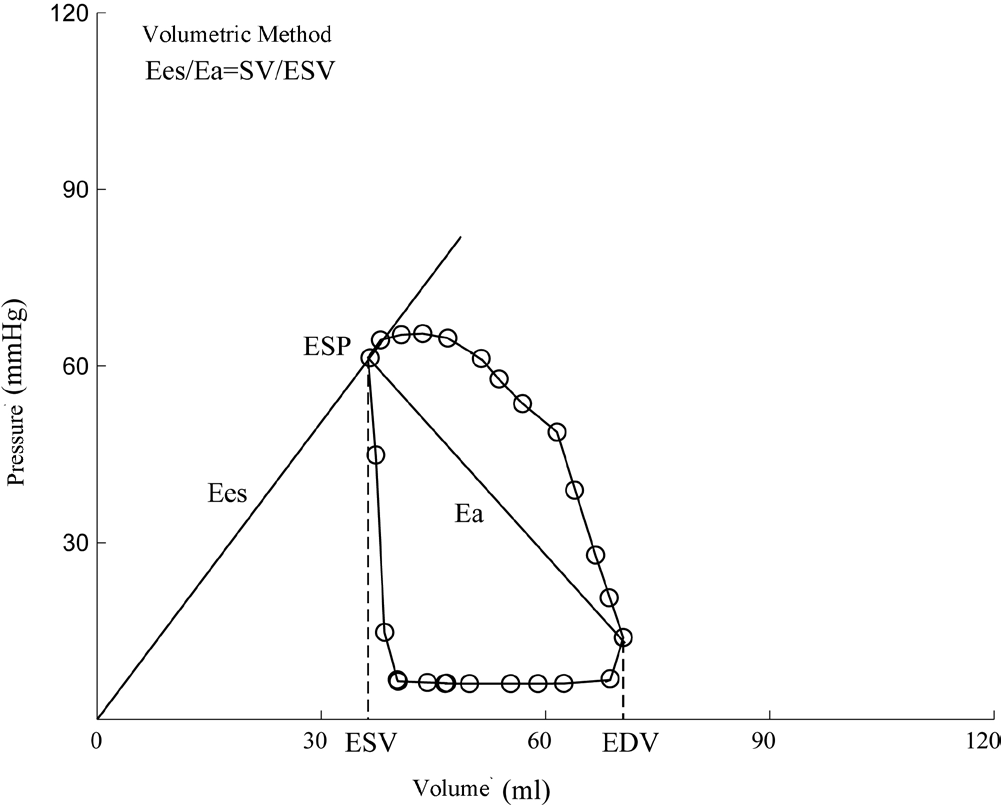
Measure *E*_es_/*E*_a_ ratio by volumetric method combined with CMR technique. Ea = arterial elasticity, Ees = end-systolic elasticity

The 4-chamber, 3-chamber and 2-chamber CINE images as well as CINE image from the basal segment till the apical segment in the right ventricle of a standard heart were obtained using CMR examination. After scanning, the images were imported to CVI 42 software workstation (circle cardiovascular imaging 42, Canada) for volumetric quantitative analysis. The ventricle endocardial borders during the systolic and diastolic periods were manually plotted. The right ventricle end-systolic volume (ESV) and end-diastolic volume (EDV) are obtained from the CINE image of the right ventricle short axis, while ESV was corresponded to ESP and EDV corresponded to EDP. The systolic pulmonary artery flow can be obtained by short-axis VENC image of the pulmonary artery, thus obtaining the SV. Among them, *E*_es_ = ESP/ESV, *E*_a_ = ESP/SV, and therefore, *E*_es_/*E*_a_ (*V*) = SV/ESV (Fig. 2).

#### 2.2.3. Pressure–volume loop.^[10]^

**Figure 3.**
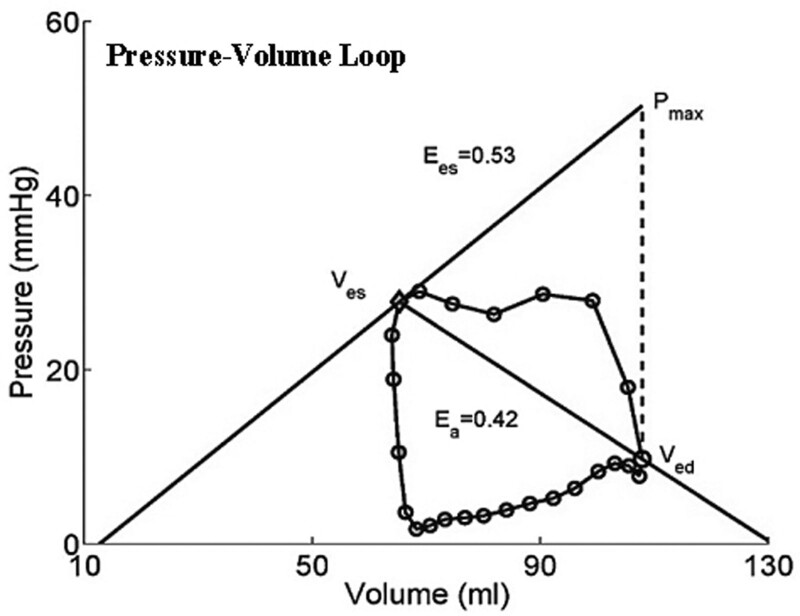
Measure *E*_es_/*E*_a_ ratio by a single pressure–volume loop obtained by the catheter. *E*_a_ = arterial elasticity, *E*_es_ = end-systolic elasticity.

Evaluation was performed using a single pressure–volume loop obtained by the catheter (Fig. 3). Assuming that the coordinates of the maximum point of *P*/*V* were (*P*_m_, *V*_m_), and the coordinates of the minimum point of *P*/*V* were (*P*_n_, *V*_n_), then *E*_es_ was considered as the slope of line that connects the maximum point of *P*/*V* and the *P*_max_. *V*_0_ was the intercept of the line with volume axis. *E*_a_ was the slope of the line that connects the maximum point of *P*/*V* and the minimum point of *P*/*V*, thus *E*_es_/*E*_a_ (*PV*) = (*P*_max_ – *P*_m_)(*V*_n_ – *V*_m_)/(*V*_max_ – *V*_m_)(*P*_m_ – *P*_n_).

### 2.3. Statistical analysis

Statistical analysis was performed for all data using SPSS19.0 software. Continuous data that met normal distribution were expressed as means ± standard deviation, while categorical data were presented by rate or composition ratio. Spearman correlation analysis was used to determine the relationship of *E*_es_/*E*_a_, NYHA classification and NP-proBNP as measured by the single beat estimation, the volume method and the pressure–volume loop method. In addition, a regression equation was established using linear regression.

## 3. Results

### 3.1. Basic clinical data

A total of 49 pulmonary hypertension patients were enrolled in our study, which included 14 males and 35 females with an average age of 31 ± 12 years. The 6-minute walking distance was 400 ± 120 m. There were 2 cases of NYHA grade I, 30 cases of grade II, 14 cases of grade III and 3 cases of grade IV. NT-proBNP was 1044.00 (181.80, 2473.00). The mean pulmonary artery pressure (mPAP) was 61 ± 19 mmHg. There were 9 patients with idiopathic pulmonary arterial hypertension, 36 cases with congenital heart disease-associated pulmonary arterial hypertension and 4 cases with CTD-associated pulmonary arterial hypertension (Table 1).

**Table 1:** Basic clinical data.

Clinical data (n = 49)	
Gender (male/female)	14/35
Age (yr)	31 ± 12
Weight (kg)	52 ± 13
Height (cm)	159 ± 11
BSA (cm^2^)	1.49 ± 0.22
6-MWD (m)	400 ± 120
HR (bpm)	86 ± 14
**RV systolic function-NYHA**	
I	2 (4.1%)
II	30 (61.2%)
III	14 (28.6%)
IV	3 (6.1%)
NT-proBNP	1044.00 (181.80, 2473.00)
**Hemodynamic parameter**	
MPA (mmHg)	94 ± 27
LPA (mmHg)	95 ± 28
RPA (mmHg)	95 ± 28
RASP (mmHg)	12 ± 8
RADP (mmHg)	5 ± 5
mRAP (mmHg)	9 ± 7
mPAP (mmHg)	61 ± 19

mPAP = mean pulmonary arterial pressure, NYHA = New York Heart Association.

### 3.2. Correlation analysis

#### 3.2.1. Results of single beat estimation.

**Figure 4.**
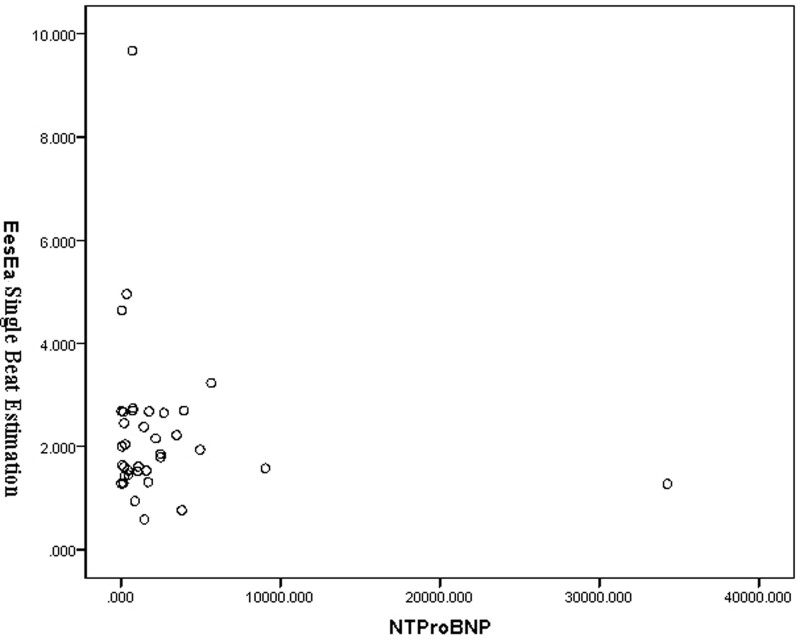
*E*_es_/*E*_a_ measured by single beat estimation was 2.07 ± 1.01. Correlation analysis showed that the results of single beat estimation were not statistically significant with NT-proBNP (*r* = –0.251). *E*_a_ = arterial elasticity, *E*_es_ = end-systolic elasticity.

*E*_es_/*E*_a_ measured by single beat estimation was 2.07 ± 1.01 (Table 2). Correlation analysis showed that the results of single beat estimation were not statistically significant with NT-proBNP (*r* = –0.251, *P* > .05) (Fig. 4), and also showed no statistical significance with NYHA cardiac function classification (*r* = –0.256, *P* > .05) (Table 3).

**Table 2:** The results of *E*_es_/*E*_a_ estimated by 3 different methods.

	Single beat estimation	Volume method	Pressure–volume loop
*E*_es_	2.28 ± 1.79	0.80 ± 0.72	2.28 ± 1.79
*E*_a_	1.15 ± 0.77	1.15 ± 0.77	0.97 ± 0.65
*E*_es_/*E*_a_	2.07 ± 1.01	0.72 ± 0.43	2.64 ± 1.48

*E*_a_ = arterial elasticity, *E*_es_ = end-systolic elasticity.

**Table 3:** The correlation analysis of *E*_es_/*E*_a_, NT-proBNP and NYHA estimated by 3 different methods.

*E*_es_/*E*_a_	NT-proBNP (*P* value)	Classification of NYHA (*P* value)
Single beat estimation	–0.439 (.002)	–0.340 (.017)
Volume method	–0.251 (.081)	–0.256 (.076)
Pressure–volume loop	–0.060 (.684)	–0.119 (.414)

*E*_a_ = arterial elasticity, *E*_es_ = end-systolic elasticity, NYHA = New York Heart Association.

#### 3.2.2. Results of pressure–volume loop.

**Figure 5.**
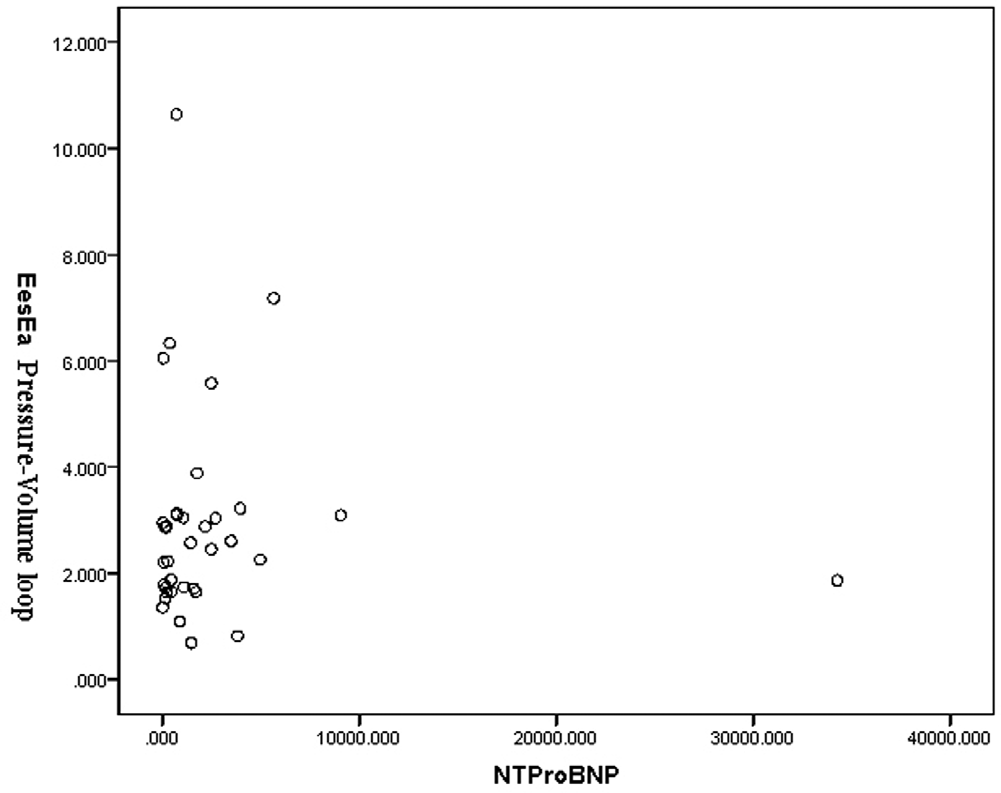
*E*_es_/*E*_a_ measured by pressure–volume loop was 2.64 ± 1.48, and the correlation analysis showed no statistical significance with NT-proBNP (*r* = –0.119). *E*_a_ = arterial elasticity, *E*_es_ = end-systolic elasticity.

*E*_es_/*E*_a_ measured by pressure–volume loop was 2.64 ± 1.48 (Table 2), and the correlation analysis showed no statistical significance with NT-proBNP (*r* = –0.060, *P* > .05) (Fig. 5). Correlation analysis with NYHA cardiac function classification showed no statistical significance (*r* = –0.119, *P* > .05) (Table 3).

#### 3.2.3. Results of volume method.

**Figure 6.**
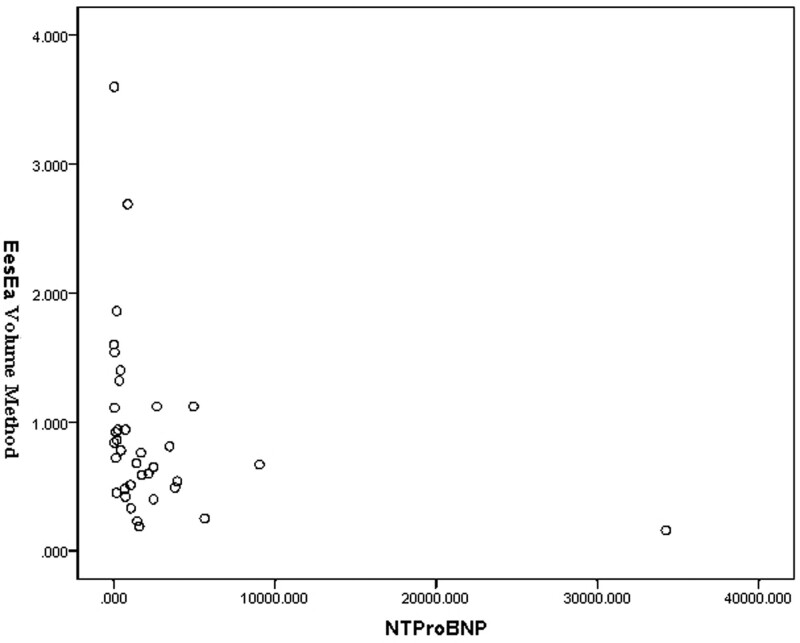
*E*_es_/*E*_a_ measured by volume method was 0.72 ± 0.43, which was significantly correlated with NT-proBNP (*r* = –0.439). *E*_a_ = arterial elasticity, *E*_es_ = end-systolic elasticity, NYHA = New York Heart Association.

*E*_es_/*E*_a_ measured by volume method was 0.72 ± 0.43 (Table 2), which was significantly correlated with NT-proBNP (*r* = –0.439, *P* < .05) (Fig. 6). They were negatively correlated, and *E*_es_/*E*_a_ was decreased with increased NT-proBNP. Correlation analysis with NYHA cardiac function classification also showed a significant negative correlation (*r* = –0.340, *P* < .05). *E*_es_/*E*_a_ was decreased with increased NYHA cardiac function classification (Table 3).

#### 3.2.4. Regression analysis of *E*_es_/*E*_a_ and log (NT-proBNP) measurement by volume method (Fig. 7 ).

Since the magnitude of NT-proBNP was greatly different from *E*_es_/*E*_a_, the log conversion was performed for NT-proBNP in our study. Correlation analysis showed that the correlation between *E*_es_/*E*_a_ and NT-proBNP as measured by volume method was statistically significant. Therefore, linear regression was applied in our study to establish a regression equation between *E*_es_/*E*_a_ and log(NT-proBNP) measured by the volume method was *Y* = –0.257*X* + 1.45. Moreover, the regression equation was statistically significant (*R* = 0.477, *P* = .001).

**Figure 7.**
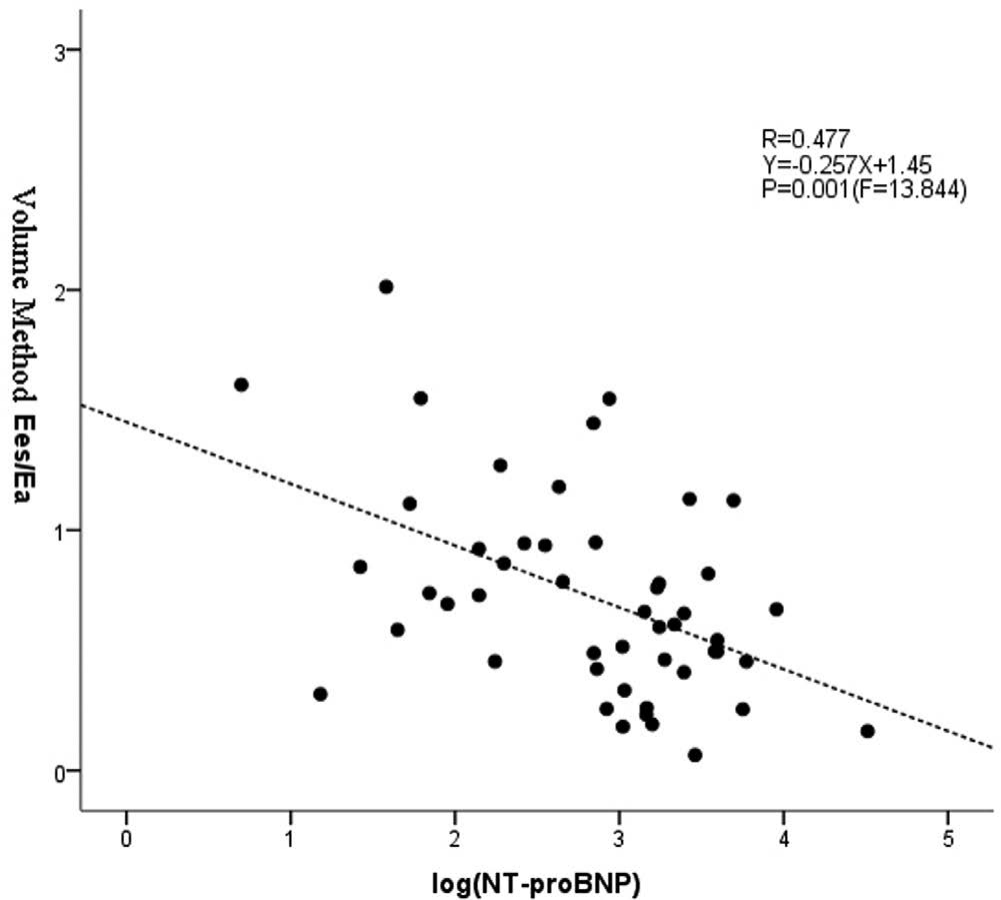
Regression analysis of *E*_es_/*E*_a_ and log (NT-proBNP) measurement by volume method. The linear regression was applied to establish a regression equation between *E*_es_/*E*_a_ and log (NT-proBNP) measured by the volume method was *Y* = –0.257*X* + 1.45. And, the regression equation was statistically significant (*R* = 0.477). *E*_a_ = arterial elasticity, *E*_es_ = end-systolic elasticity.

## 4. Discussion

Right ventricle function is the main determining factor in patients with severe pulmonary arterial hypertension, associating with the exercise tolerance and survival rate which has been recognized by most of the researchers.^[6,11]^ The single beat estimation, volume method combined with CMR technique and pressure–volume loop were used for quantitative evaluation of the right ventricle function in clinical practice. The right ventricle-pulmonary artery coupling *E*_es_/*E*_a_ ratio can be obtained by these 3 methods, and the ratios were different due to the usage of different methods for calculating *E*_es_/*E*_a_. The volume method combined with CMR technique produces the simplest right ventricle-artery coupling index (*E*_es_/*E*_a_) based on the CMR images of the end-systolic volume and end-diastolic volume, and thus it has been gradually applied in clinical practice. Some studies have demonstrated that^[12,13]^ the result of right ventricle-artery coupling index that is obtained by the volume method combined with CMR technique is superior when compared to other methods. The method not only ensures the coupling effect of right ventricle systolic function on the after load response, but also has prognostic significance. Moreover, it is noninvasive and easy to apply.

In this study, *E*_es_/*E*_a_ ratios obtained by the 3 methods were greatly different. The single beat estimation was consistent with pressure–volume loop in measuring *E*_es_, thus *E*_es_ data were the same in both these methods. However, due to different measurement methods of *E*_a_, the final *E*_es_/*E*_a_ ratios obtained were different. *E*_es_/*E*_a_ ratio was the largest measurement obtained by pressure–volume loop, while it was the smallest measurement obtained by the volume method combined with CMR technique (Table 2). Further analysis showed that *E*_es_/*E*_a_ as measured by the volume method combined with CMR technique was not only correlated with NT-proBNP, but also related to NYHA cardiac function classification. Moreover, linear regression equation (*y* = –0.257*x* + 1.45) was established with logNT-proBNP, suggesting that *E*_es_/*E*_a_ ratio was decreased with increased right ventricle function. However, *E*_es_/*E*_a_ ratios obtained by single beat estimation and pressure–volume loop were not correlated with NT-proBNP and NYHA cardiac function classification as well as logNT-proBNP. This further suggested that the right ventricle-pulmonary artery coupling (*E*_es_/*E*_a_) obtained by volume method combined with CMR imaging technique for quantitative assessment of the cardiac function in patients with pulmonary arterial hypertension was considered to be a feasible and reliable method. These results were far superior to the invasive data as measured by the right cardiac catheter. However, since this study included only adult patients with pulmonary arterial hypertension, there are no reliable data to support that the volume method combined with the CMR imaging technique was feasible for pediatric patients with pulmonary arterial hypertension. A study^[14]^ that used CMR to perform quantitative assessment for the right heart function in children with pulmonary arterial hypertension confirmed that CMR is a noninvasive and feasible method. Therefore, along with our study, it fully demonstrated that the volume method combined with CMR technique was a reliable noninvasive quantitative method for evaluating the right heart function in adult as well as pediatric patients with pulmonary arterial hypertension. This in turn provided reliable data for the clinical treatment and prognosis. The single beat estimation and pressure–volume loop can obtain *E*_es_/*E*_a_ by combining with the catheter method, which are also considered as important methods for quantitative evaluation of the development of right heart function in pulmonary arterial hypertension. However, the assumption that the shape of the pressure–volume loop was obtained based on the catheter has large effect on calculating the result of *E*_es_. Moreover, the formula for calculating *E*_es_/*E*_a_ remains complicated, and its accuracy was affected by many factors. In this study, single beat estimation and pressure–volume loop were not correlated with NT-proBNP, logNT-proBNP and NYHA cardiac function classification, confirming that the accuracy of these methods were greatly affected by variables in clinical practice. Moreover, the patients would suffer from the invasive puncturing process during data acquisition using the catheter, highlighting the role of noninvasive and accurate volume method combined with CMR technique in future clinical application.

## 5. Conclusion

In summary, this study confirmed that the right ventricle-pulmonary artery coupling can be obtained by volume method combined with CMR imaging technique. This method provided an accurate and reliable *E*_es_/*E*_a_ ratio, confirming the reliability and non-invasiveness of the method for quantitative assessment of right heart function of patients with pulmonary arterial hypertension. However, this conclusion should be further verified in a larger patient population.

## 6. Study limitation

The results of this study were limited by the small number of patients with PAH and short follow-up. Hence, more patients with PAH, multiple centers, and a longer follow-up might be needed to validate the conclusion of the present study. In addition, animal and clinical trials may be required to explore the definition and efficacy of the noninvasive methods to measure the right ventricular function, which may be used in the clinic to improve life expectancy and quality of patients with PAH.

## Author contributions

**Conceptualization:** Yaling Dong, Yu Li.

**Data curation:** Laichun Song.
